# Supplementary material for: Phenotypic plasticity and genetic control in colorectal cancer evolution
Source: Nature. 2022 Oct 26;611(7937):744–53. doi: 10.1038/s41586-022-05311-x (PMC9684078; doi:10.1038/s41586-022-05311-x)
Supplement: Supplementary file 1 — Supplementary Figs. 1–27. [file 41586_2022_5311_MOESM1_ESM.pdf]

nature portfolio

https://doi.org/10.1038/s41586-022-05311-x

Supplementary information

Phenotypic plasticity and genetic control in colorectal cancer evolution

In the format provided by the authors and unedited

Supplementary Figures

Phenotypic plasticity and genetic control in colorectal cancer evolution

Jacob Househam[1,2,*], Timon Heide[1,3,*], George D Cresswell[1], Inmaculada Spiteri[1], Chris Kimberley[2], Luis Zapata[1], Claire Lynn[1], Maximilian Mossner[1,2], Javier Fernandez-Mateos[1], Alessandro Vinceti[3], Ann-Marie Baker[1,2], Calum Gabbutt[1,2], Alison Berner[2], Melissa Schmidt[2], Bingjie Chen[1], Eszter Lakatos[1,2], Vinaya Gunasri[1,2], Daniel Nichol[1], Helena Costa[4], Miriam Mitchinson[5], Daniele Ramazzotti[6], Benjamin Werner[2], Francesco Iorio[3], Marnix Jansen[4], Giulio Caravagna[1,7], Chris P. Barnes[8], Darryl Shibata[9], John Bridgewater[4], Manuel Rodriguez-Justo[4], Luca Magnani[10], Andrea Sottoriva[1,3,†], and Trevor A. Graham[1,2,†]

[1]Centre for Evolution and Cancer, The Institute of Cancer Research, London, UK
[2]Centre for Genomics and Computational Biology, Barts Cancer Institute, Queen Mary University of London, London, UK
[3]Computational Biology Research Centre, Human Technopole, Milan, Italy
[4]UCL Cancer Institute, University College London, London, UK
[5]Histopathology Department, University College London Hospitals NHS Foundation Trust, London, UK
[6]Department of Medicine and Surgery, University of Milano-Bicocca, Milan, Italy
[7]Department of Mathematics and Geosciences, University of Triest, Triest, Italy
[8]Department of Cell and Developmental Biology, University College London, London, UK
[9]Department of Pathology, University of Southern California Keck School of Medicine, Los Angeles, CA, 90033, USA
[10]Department of Surgery and Cancer, Imperial College London, London, UK
[*]equal contribution
[†]Correspondence to: andrea.sottoriva@fht.org and trevor.graham@icr.ac.uk

Contents

1 **Normal colon scRNA-seq** **3**

2 **Phylogenetic signal** **4**

3 **Expression clustering** **8**

4 **Exploring eQTL results** **13**

5 **eQTL and MSI** **16**

6 **Mutations and phylogenetics** **19**

7 **Selection inference** **22**

8 **Combining analyses** **24**

List of Supplementary Figures

S1 Expression of gene list in normal colon scRNA-seq 3
S2 Expression of gene groups in normal colon scRNA-seq 3
S3 Explanation of phylogenetic signal analysis 4
S4 Number of phylogenetic genes versus number of samples 4
S5 Impact of RNA-seq normalisation method on phylogenetic signal analysis 5
S6 Phylogenetic signal in colorectal cancer with purity-adjusted expression. 6
S7 Assessment of power to detect phylogenetic signal for multi-region tumours 7
S8 Phylogenetic trees and expression-based clustering 8
S9 Intermixing scores comparison 9
S10 Expression clustering with heatmaps 10
S11 Permutation test for correlations between gene expression and sample region-of-origin 11
S12 Analysis of the impact of immune infiltration on expression differences 12
S13 Investigation of genes negatively correlated with copy number 13
S14 eQTL validation using Hartwig mCRC cohort 14
S15 Post-hoc power analysis of eQTLs 15
S16 Principal component analysis of germline SNPs 16
S17 QQ plot between MSS and MSI eQTL analyses 16
S18 eQTL results with MSI added as a cofactor 17
S19 Comparing R^2 values between original and MSI co-factor analyses 18
S20 Gene essentiality analysis with CRISPR screens 19
S21 High resolution BaseScope® image C539 20
S22 High resolution BaseScope® image C537 20
S23 Measurements of subclonal intermixing for each tumour 21
S24 Effect of mutation rate and peripheral growth on simulations 22
S25 Transcriptional and epigenetic changes in selected clones 22
S26 Gene set enrichment analysis of gene expression changes in subclones 23
S27 Assessment of heritable changes associated with subclonal selection. 24

1 Normal colon scRNA-seq

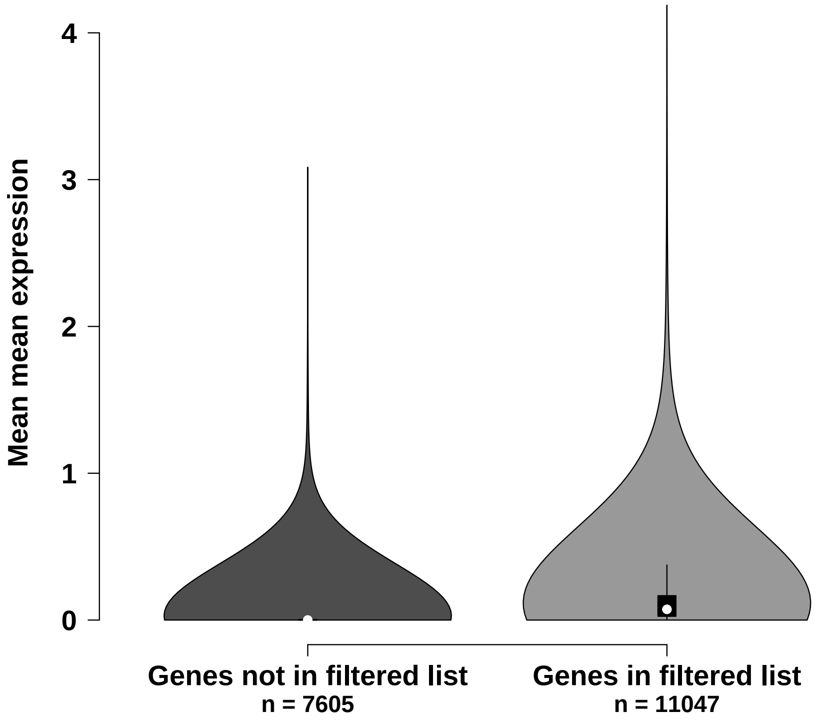

Supplementary Figure S1: Violin plot showing that genes identified as moderately/highly expressed in colon cancer glands are more highly expressed in normal colon cells from healthy adults than all other genes. Two-sided Wilcoxon signed rank test (p<1e-6).

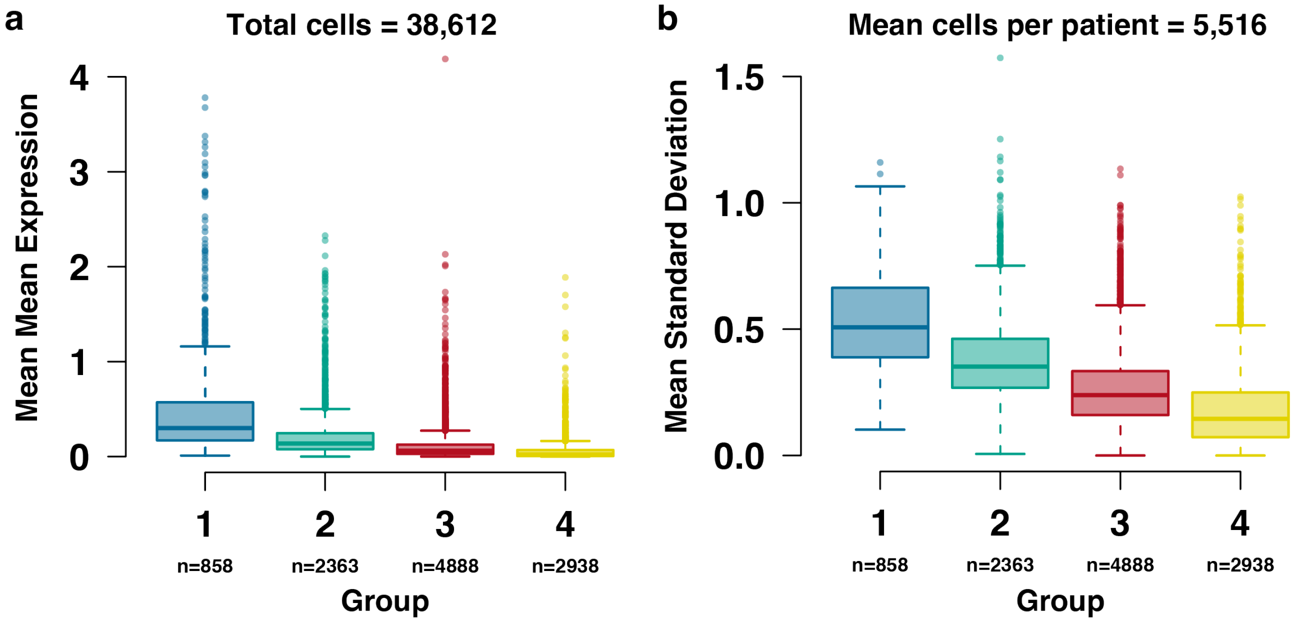

Supplementary Figure S2: Mean mean expression and mean standard deviation of genes in colonic single cells of healthy adults, split by gene group.

2 Phylogenetic signal

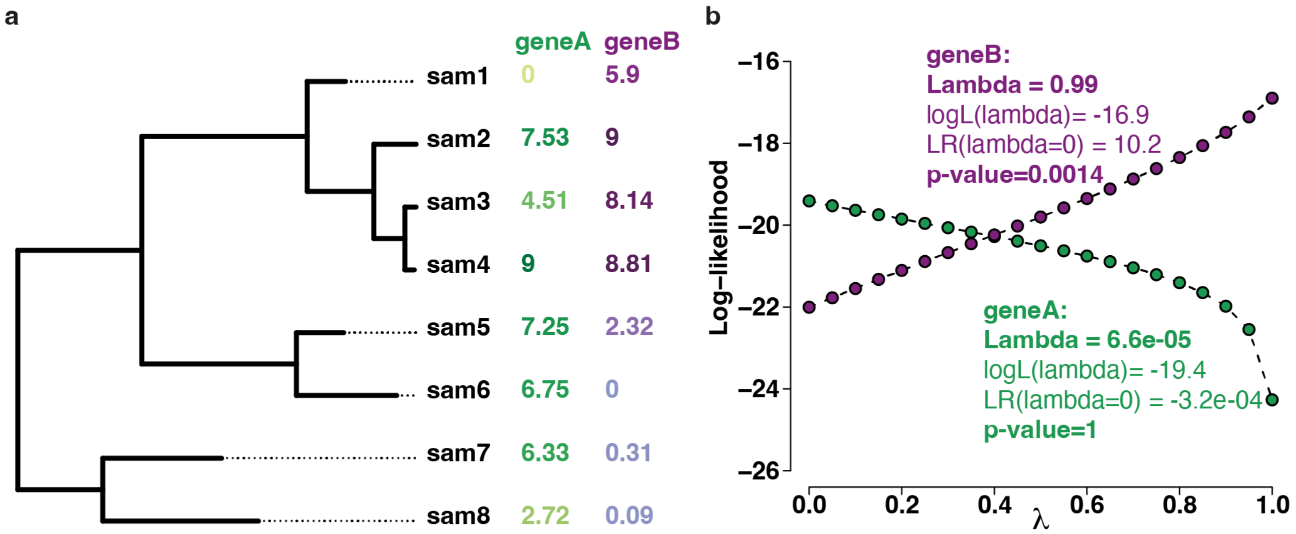

Supplementary Figure S3: Explanation of phylogenetic signal analysis. (a) Example phylogenetic tree with branch lengths and expression of two example genes shown. Gene A's expression was randomly generated for each sample, while Gene B's expression follows a random walk along the tree, meaning expression will tend to be more similar for closely related samples. Expression for both genes is scaled between 0 and 9. **(b)** Illustration of Pagel's lambda calculation: log-likelihood values are calculated for all values of lambda between 0 and 1, where a lambda of 1 means the structure of the tree itself explains the gene expression while a lambda of 0 means that the tree must lose all of its structure to explain the evolution of the gene expression under a random walk. The lambda estimate is the lambda with the maximum log-likelihood while a likelihood ratio test against lambda=0 then tests for the significance of phylogenetic signal (adjustments are not made for multiple testing).

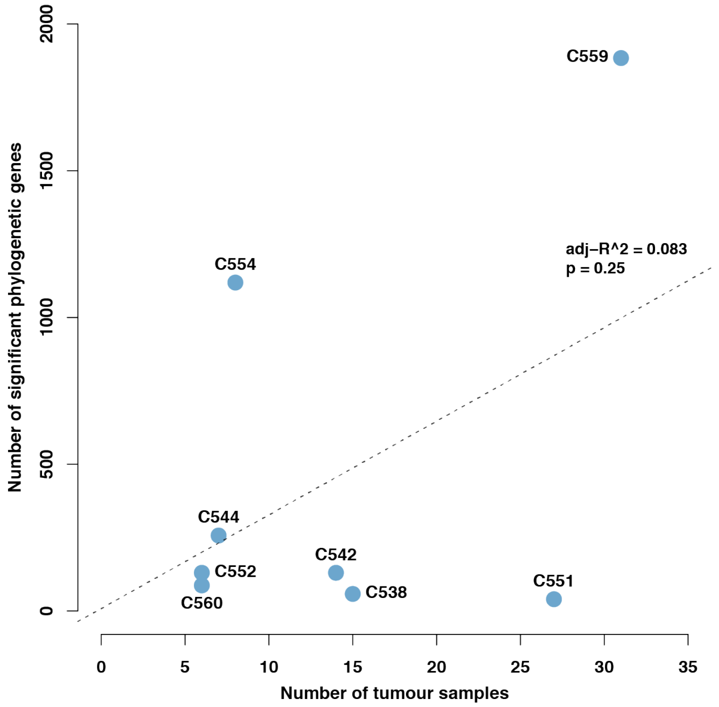

Supplementary Figure S4: Scatterplot of the number of phylogenetic genes per tumour against the number of multi-region tumour samples analysed. The detection of phylogenetic signal did not depend upon sample number (linear regression two-sided t-test).

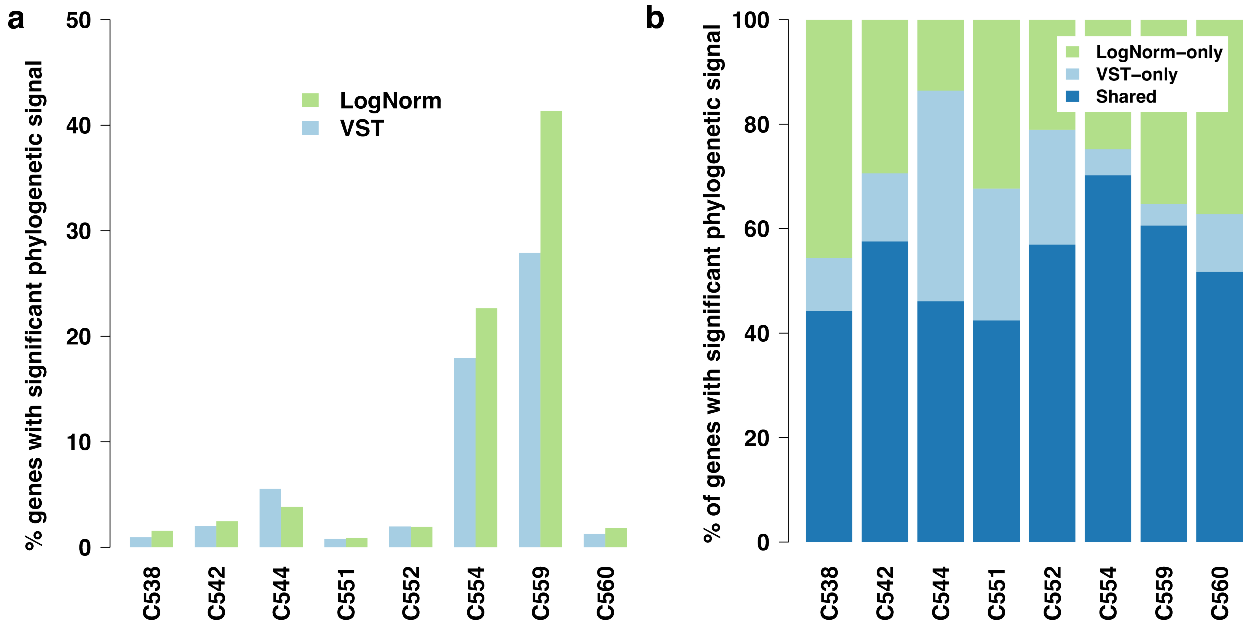

Supplementary Figure S5: The impact of RNA-seq normalisation method on phylogenetic signal analysis. **(a)** Bar chart showing the impact of RNA-seq normalisation method on the percent of genes that were found to be phylogenetic per tumour (P<0.05). **(b)** Clustered bar chart showing in each tumour, of the genes found to be phylogenetic in at least one analysis (P<0.05), the percentage that were found in both, only VST or only LogNorm respectively. VST=variance-stabilising transformation, LogNorm=log-normalisation.

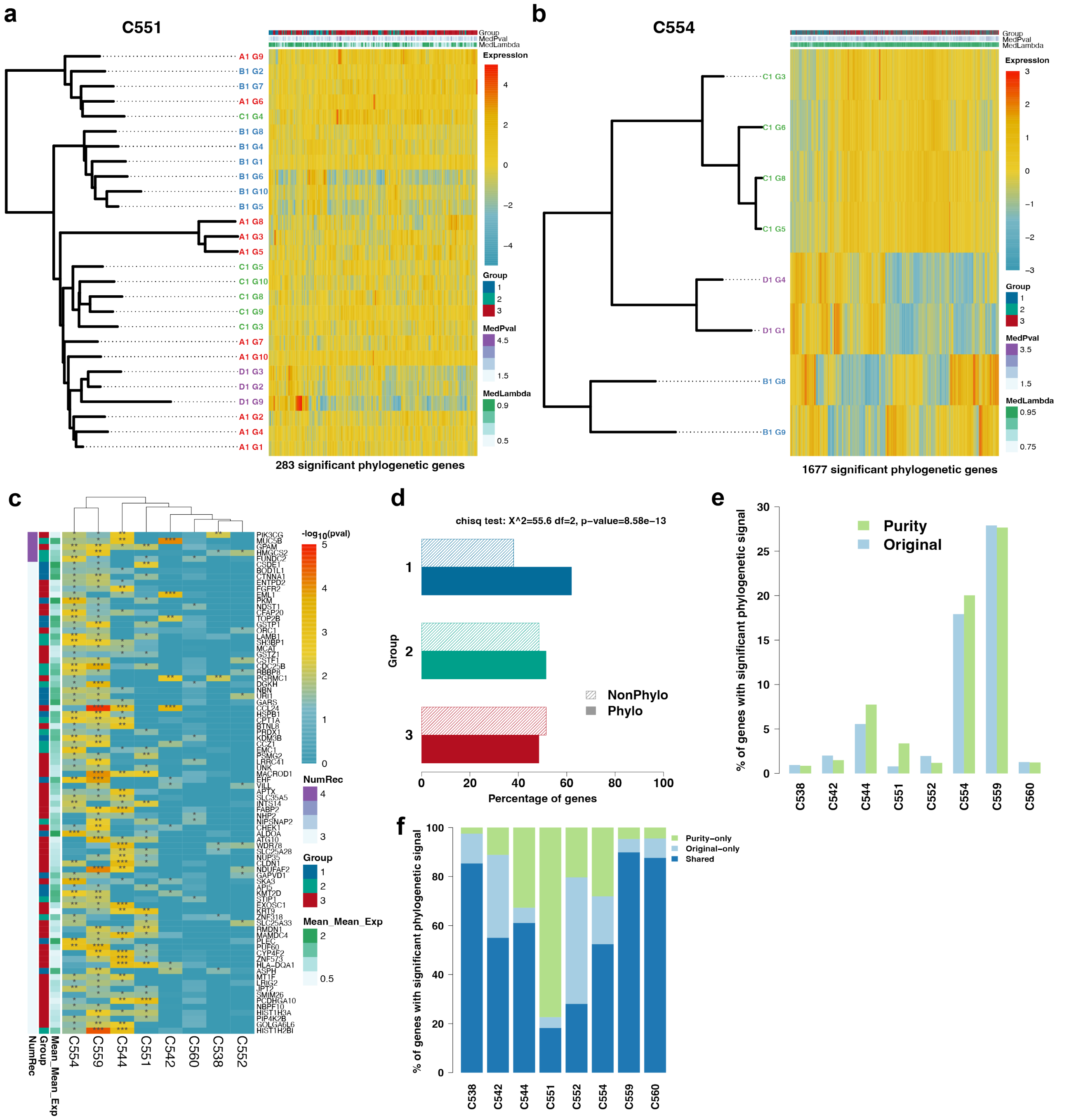

Supplementary Figure S6: Phylogenetic signal in colorectal cancer with purity-adjusted expression. **(a)** and **(b)** Phylogenetic trees and heatmaps of genes with evidence of phylogenetic signal (P<0.05) for tumours C552 and C554 respectively. **(c)** Genes with recurrent phylogenetic signal across tumours, genes shown were found to have evidence of phylogenetic signal (P<0.05) in at least three tumours **(d)** Results of two-sided chi-squared test showing whether gene groups were enriched for phylogenetic genes (genes with phylogenetic signal in at least one tumour). **(e)** Bar chart showing the impact of RNA-seq normalisation method on the percent of genes that were found to be phylogenetic per tumour. **(f)** Clustered bar chart showing in each tumour, of the genes found to be phylogenetic in at least one analysis, the percentage that were found in both, only Original or only Purity respectively.

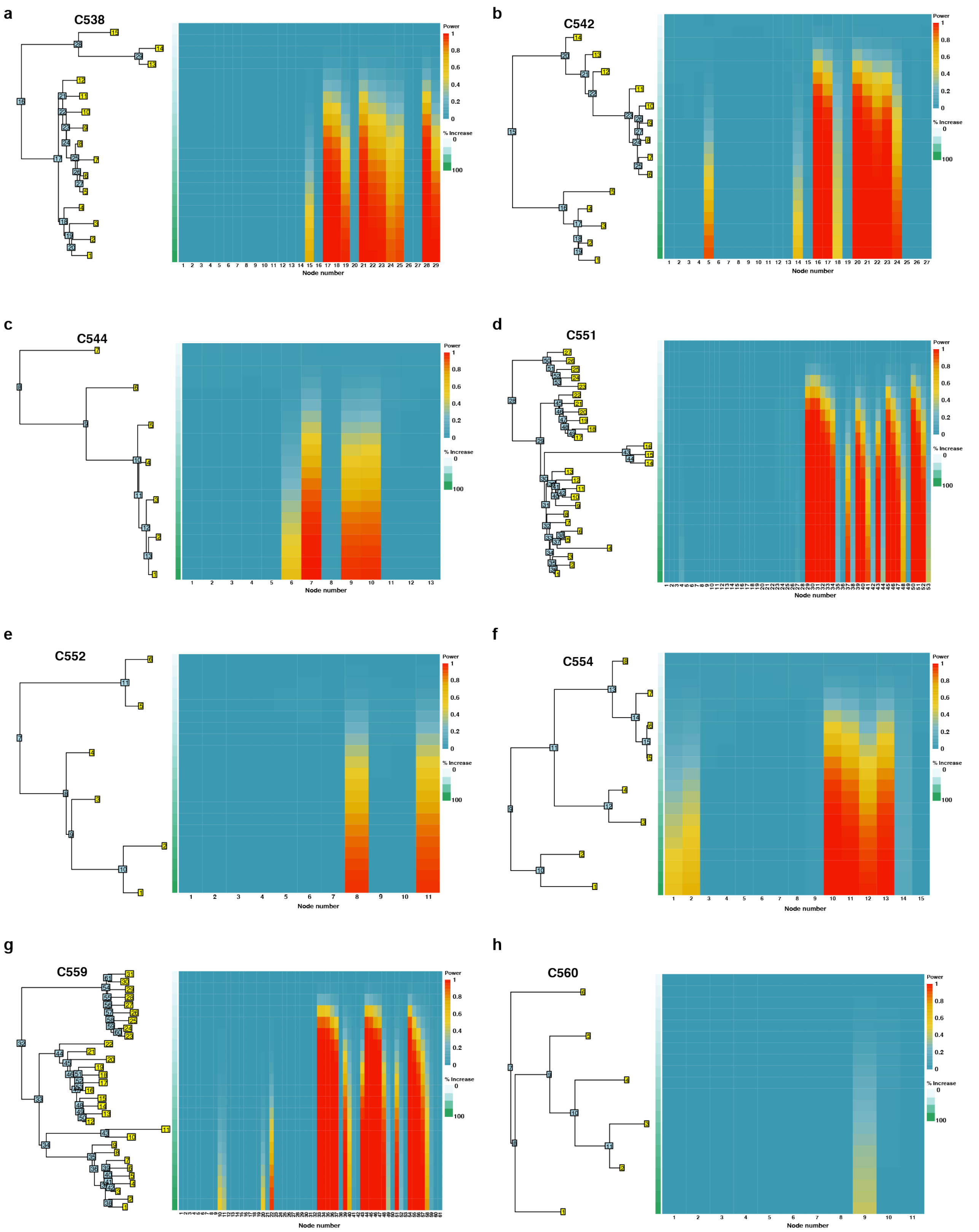

Supplementary Figure S7: Assessment of power to detect phylogenetic signal for multi-region tumours. Left: labelling of nodes for tumour phylogenetic trees. Right: Power to detect phylogenetic signal (colour) by the magnitude of expression change (rows) induced at a particular node (columns). Listed by cancer tumour: **(a)** C538. **(b)** C542. **(c)** C544. **(d)** C551. **(e)** C552. **(f)** C554. **(g)** C559. **(h)** C560.

3 Expression clustering

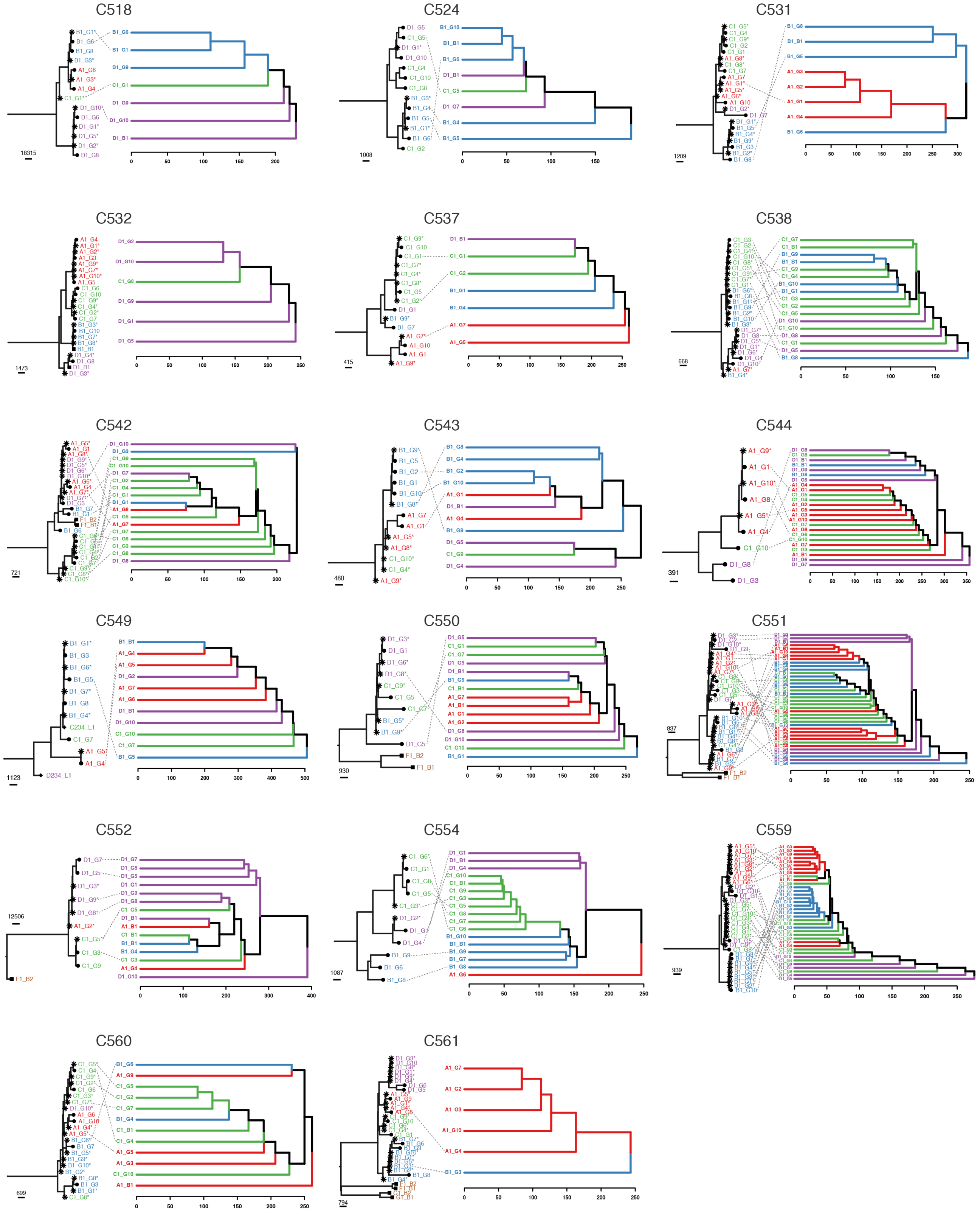

Supplementary Figure S8: WGS-based phylogenetic trees plotted side-by-side with expression-based hierarchically clustered dendrograms for the 17 tumours with at least 5 RNA-seq samples. Dotted lines show matching samples and samples are coloured according to region-of-origin.

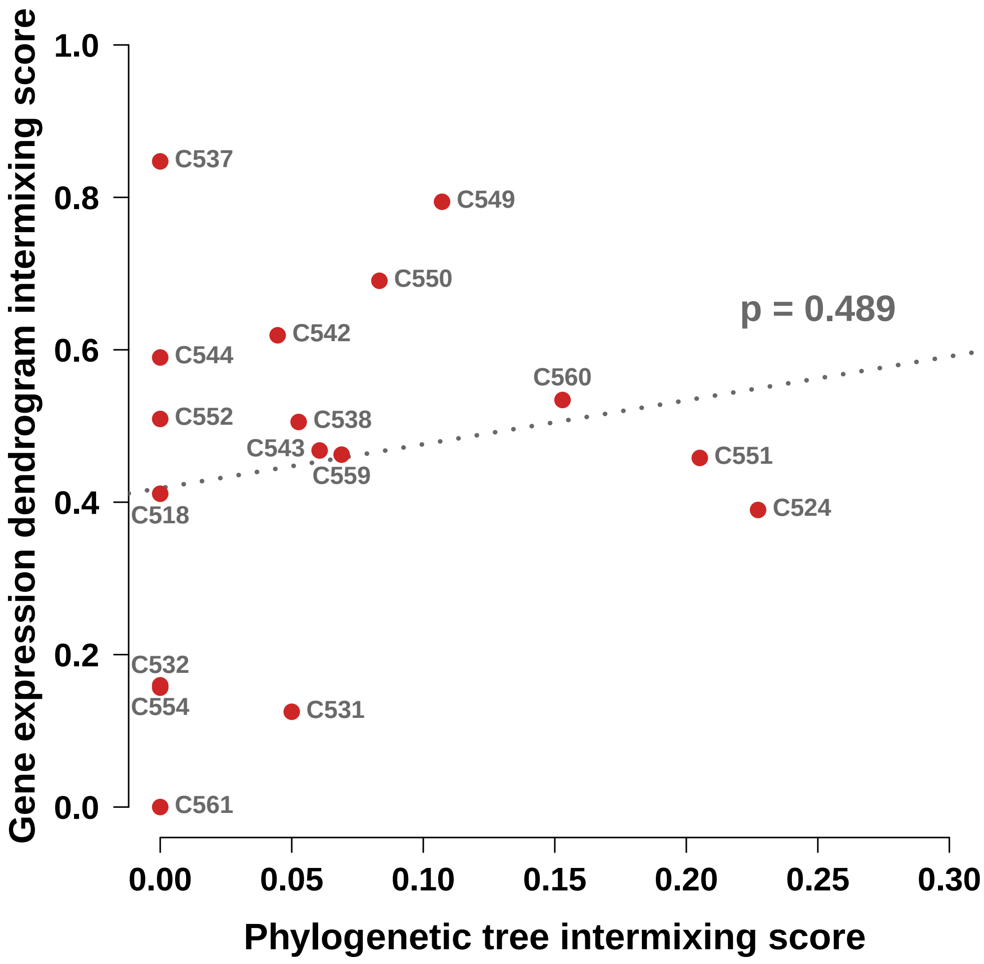

Supplementary Figure S9: Scatter plot comparing intermixing scores calculated using the WGS-based phylogenetic trees versus gene expression-based hierarchically clustered dendrograms. Linear regression two-sided t-test.

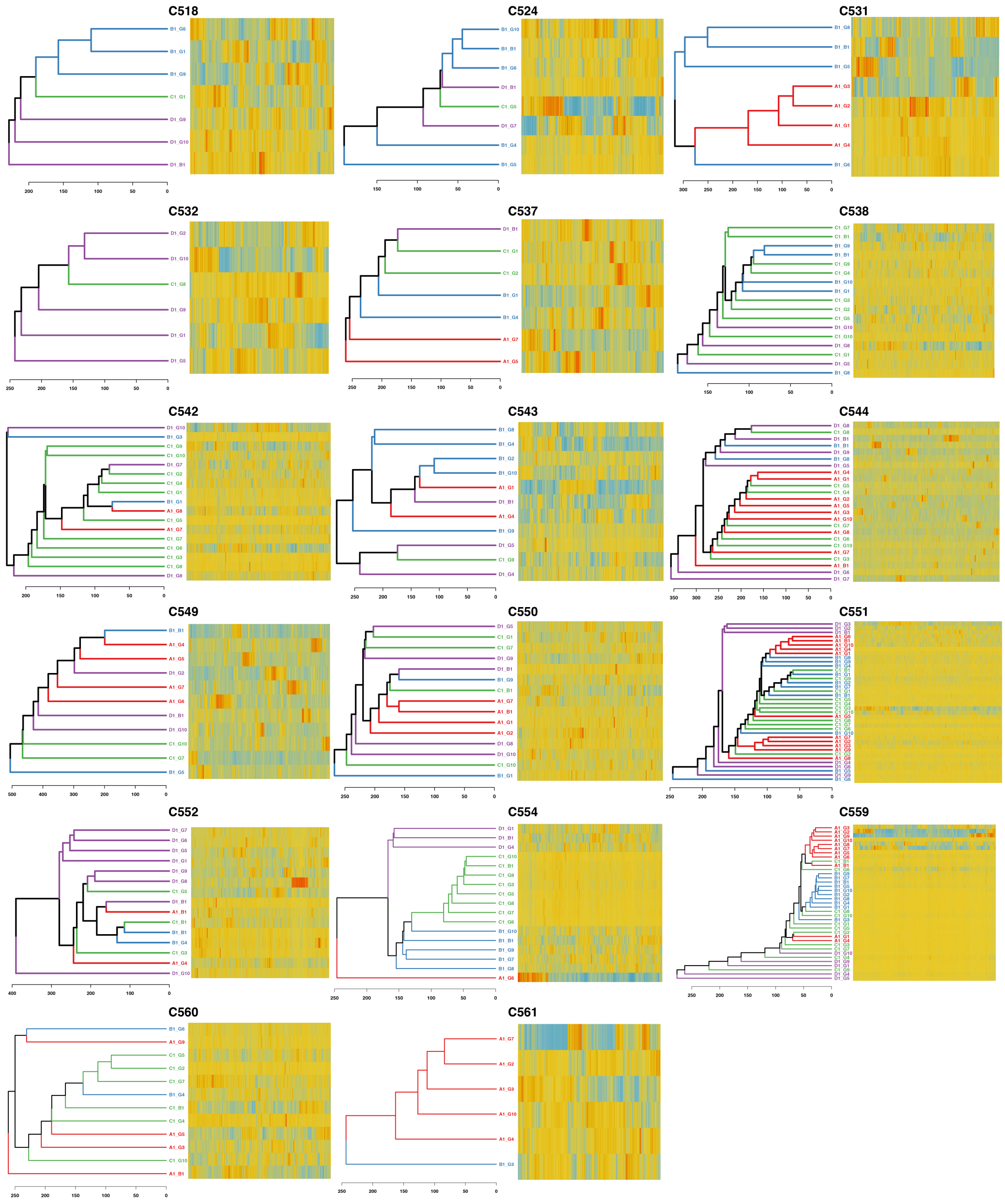

Supplementary Figure S10: Hierarchically clustered dendrograms of 17 tumours based on the expression of genes from Groups 1-3 (n=8368), with matching heatmaps. Colours of the sample names, nodes and branches of the dendrograms correspond to the tumour region; A = red, B = blue, C = Green, D = purple.

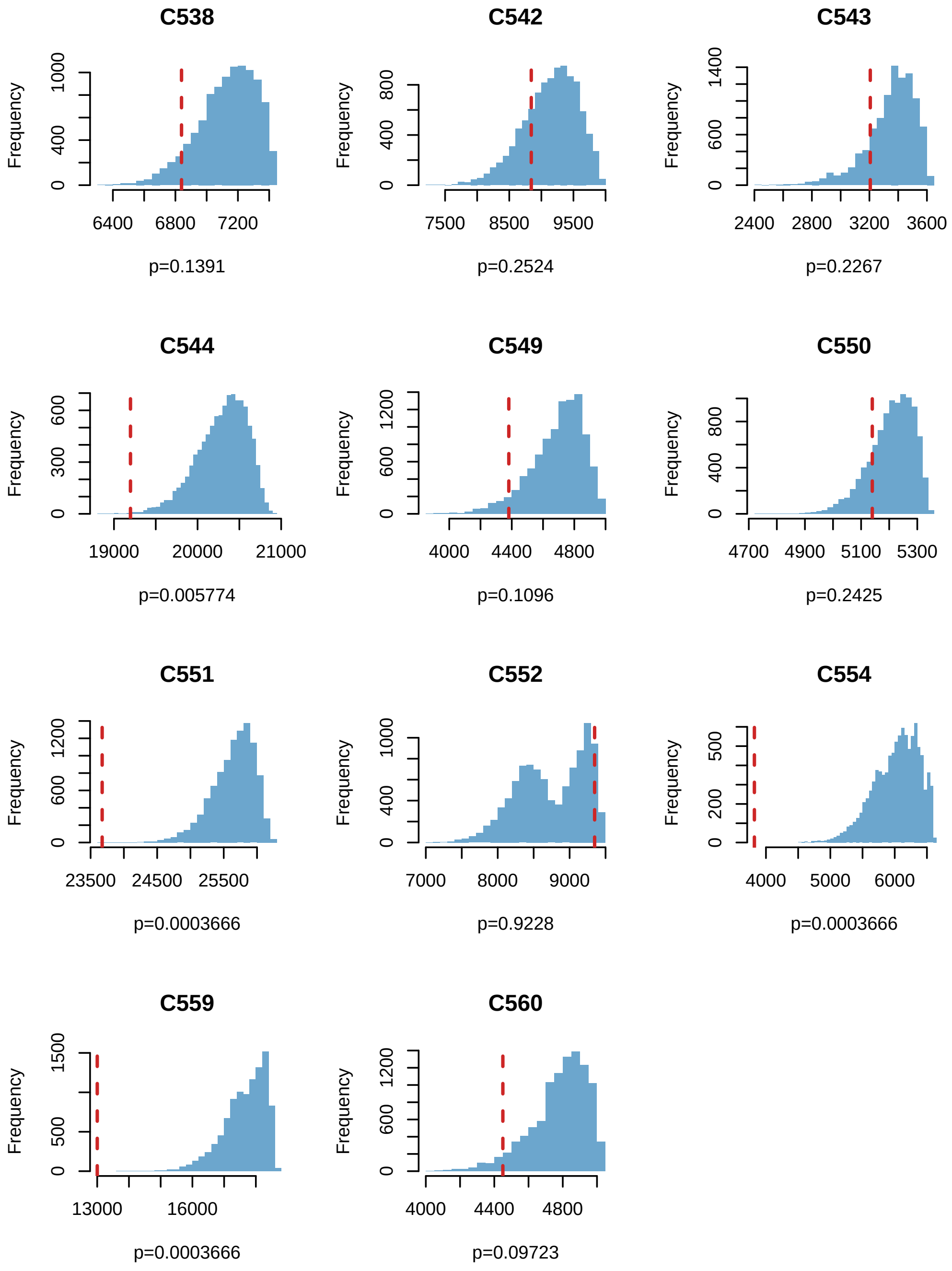

Supplementary Figure S11: Permutation test for correlations between gene expression and sample region-of-origin. Red dashed lines show the empirically observed value of the statistic, and the blue histogram the computed null distribution. Test is effectively one-sided and p-values are FDR-adjusted.

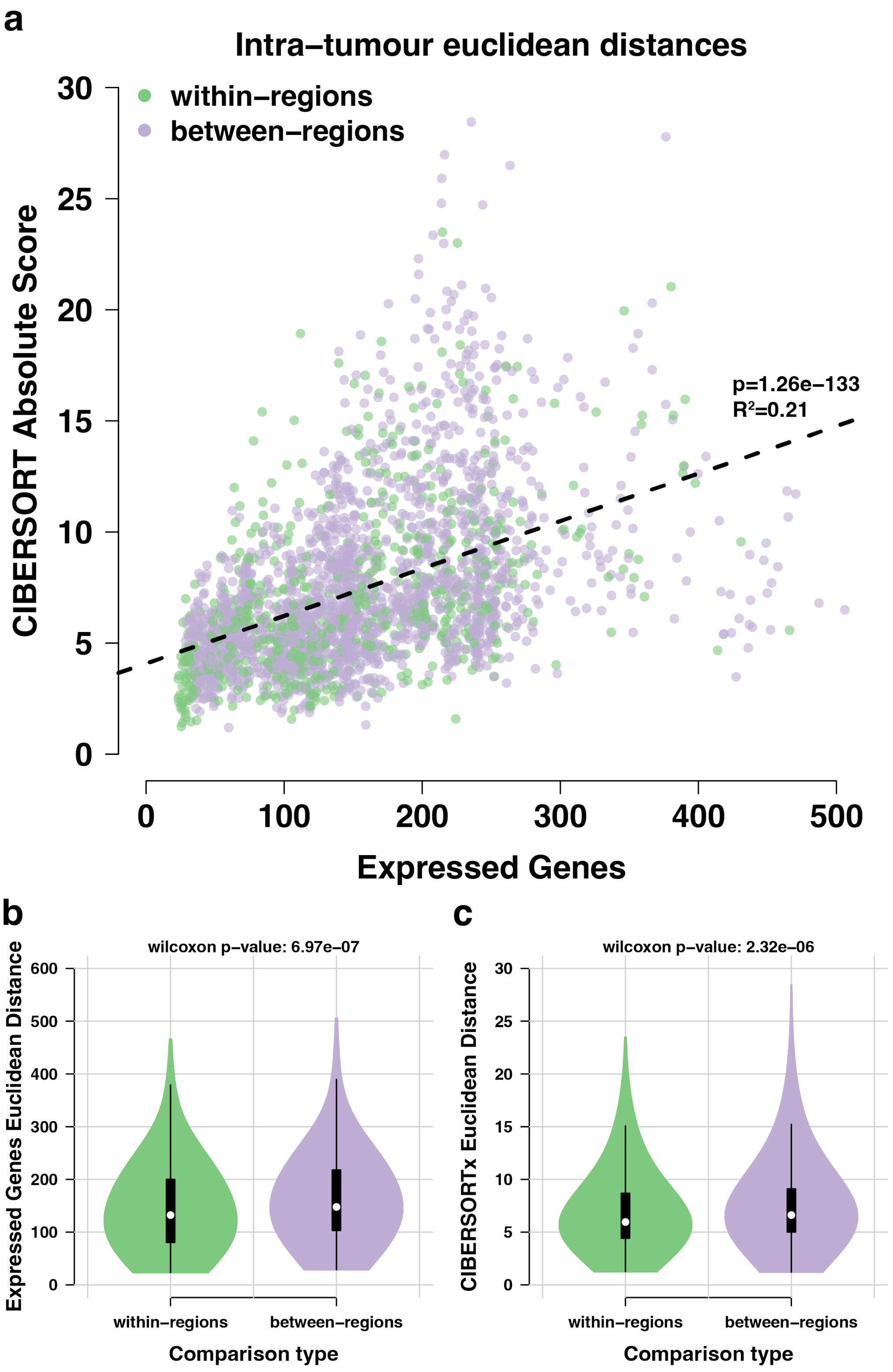

Supplementary Figure S12: Analysis of the impact of immune infiltration on expression differences. 239 samples from 17 tumours; n=2567 pairwise comparisons. **(a)** Scatter plot showing the correlation of Euclidean distances between samples when calculated from the expression of genes and CIBERSORTx estimates respectively. Each dot is a within-tumour sample pair, dots are coloured by pair type (i.e., within-region/between-region). Linear regression two-sided t-test. **(b)** and **(c)** Violin plots showing the Euclidean distance of pairwise samples split by pair type (703 within-region comparisons versus 1864 between-region comparisons, two-sided Wilcoxon signed rank tests) based on **(b)** gene expression and **(c)** CIBERSORTx estimates.

4 Exploring eQTL results

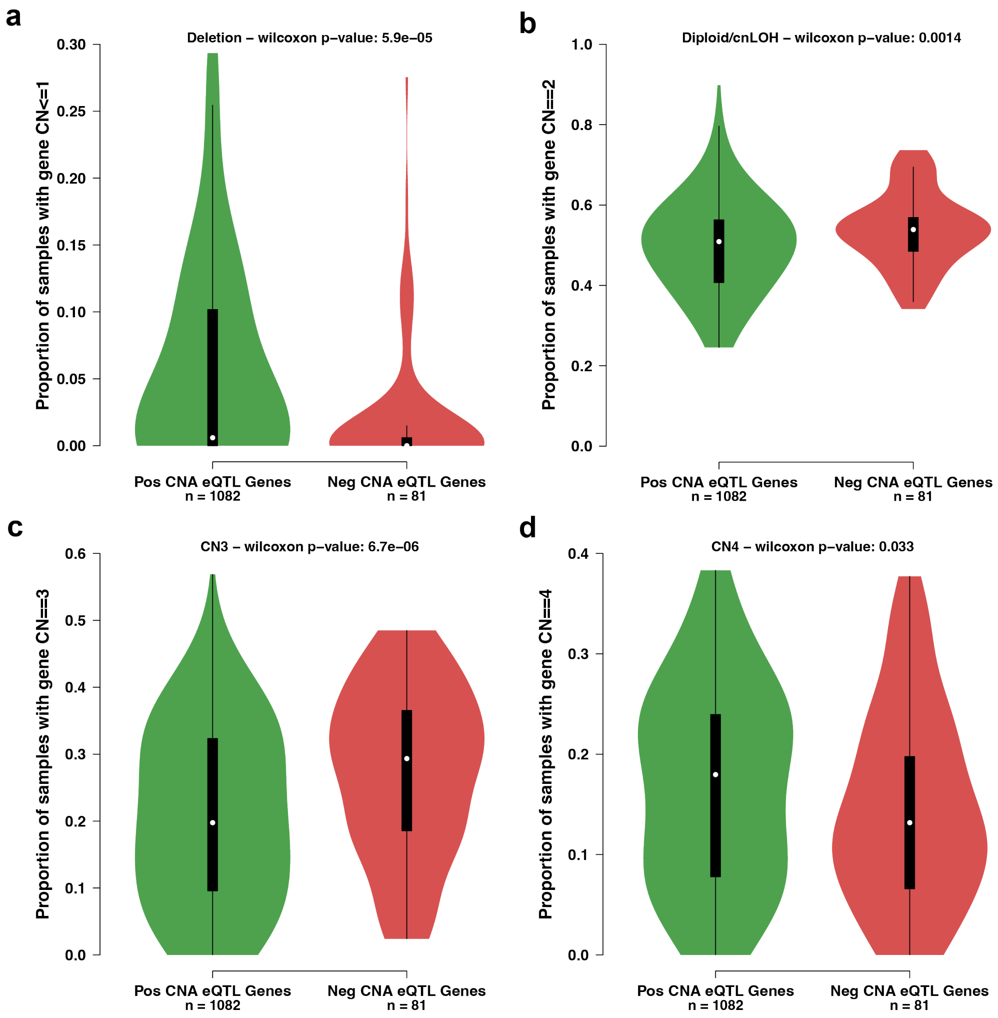

Supplementary Figure S13: Frequency of associations between gene copy number alteration and change in gene expression, by direction of correlation and average locus-specific copy number. X-axis: direction of copy number-expression correlation. Y-axis: proportion of samples across the whole cohort with specified copy number. **(a)** Genetic deletions are more commonly associated with positive correlations between gene expression and copy number. **(b)** Loci with total copy number two (which includes copy neutral loss of heterozygosity – cnLOH - events) are more likely to show a negative correlation between copy number and gene expression. **(c)** Unbalanced gains resulting in total gene copy number 3 are also associated with negative correlation between copy number and gene expression, whereas **(d)** copy number alterations resulting in total copy number 4 (typically balanced gain) are significantly associated with positive correlations with copy number.

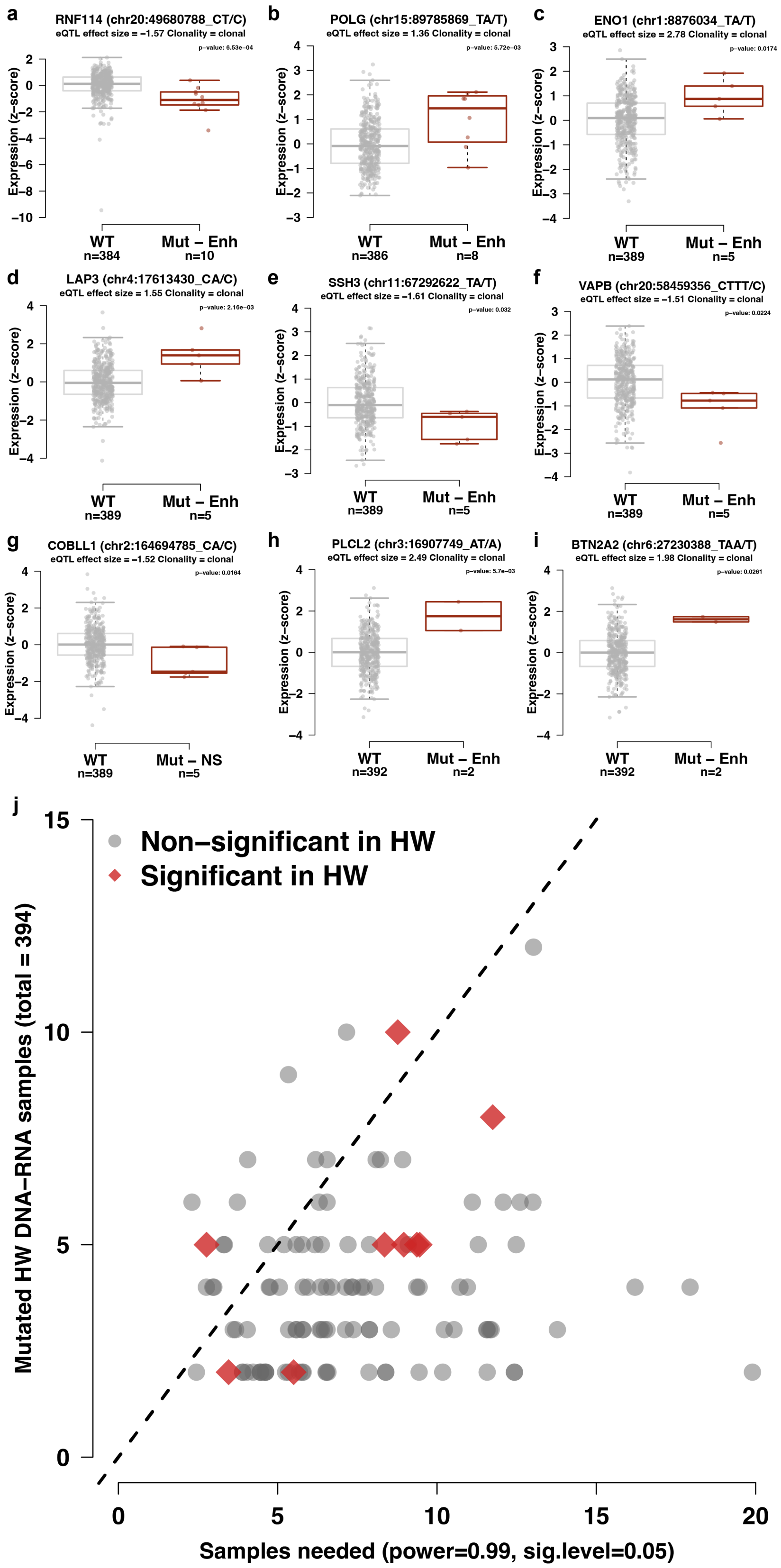

Supplementary Figure S14: Investigating gene mutation-expression correlations (eQTLs) in the Hartwig mCRC cohort. **(a-i)** Recurrent eQTLs in the Hartwig metastatic CRC tumours which also significantly correlate with expression changes in mutated samples. Linear regression two-sided t-tests, p-values are not adjusted for multiple comparisons. **(j)** Power analysis demonstrating the lack of power to detect most eQTLs in the Hartwig cohort. X-axis: Predicted number of mutated Hartwig samples required to detect effect of size observed in our cohort. Y-axis: Actual number of mutated Hartwig samples available in the cohort. Red diamonds: significant effect replicated in Hartwig, grey circles: effect not detected in Hartwig.

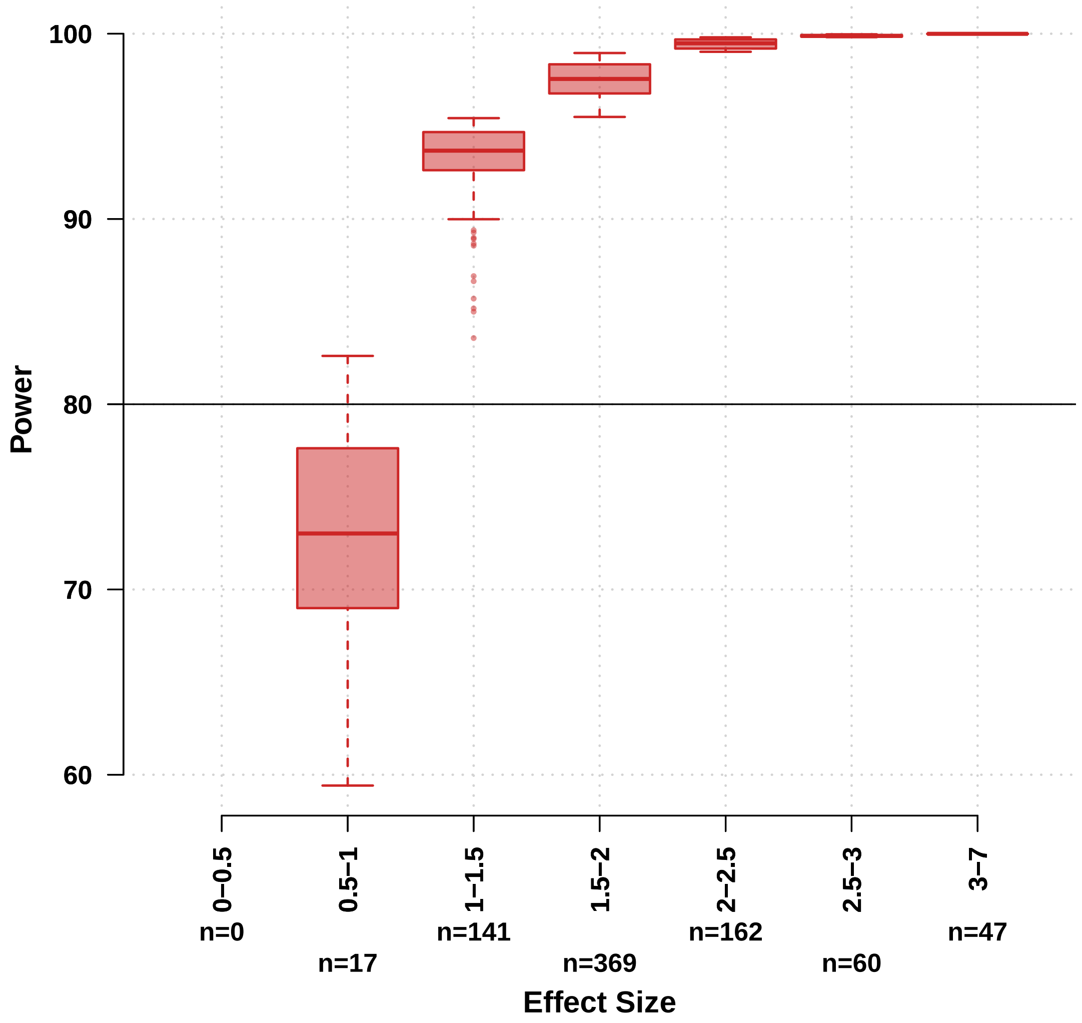

Supplementary Figure S15: Box plot showing post-hoc power analysis of mutation eQTL effect sizes. Effect sizes have been binned and the number of models in each bin are shown at the bottom of the plot.

5 eQTL and MSI

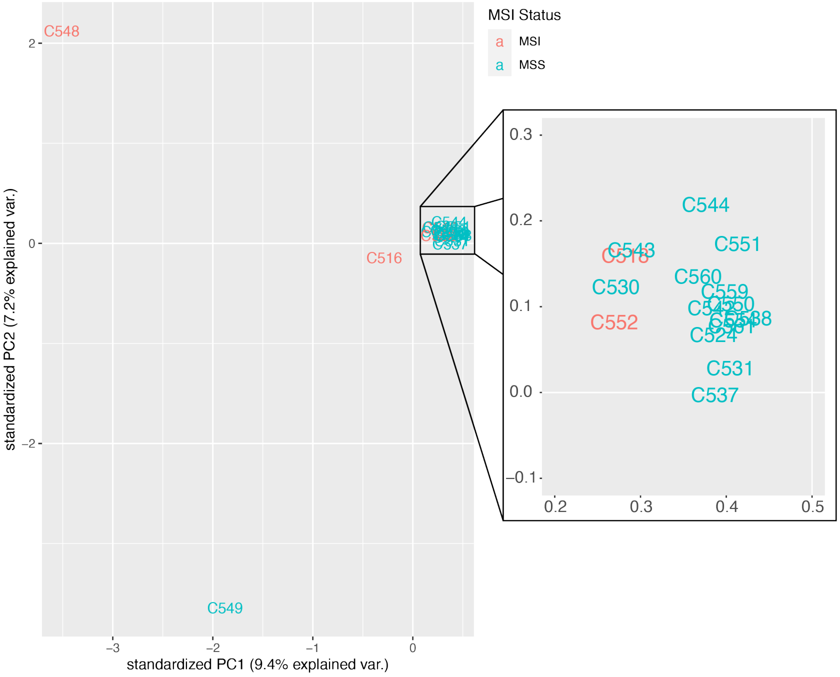

Supplementary Figure S16: Plot of the top two principal components from PCA analysis of germline SNPs in patients used in the eQTL analysis. Patients are coloured according to MSI status.

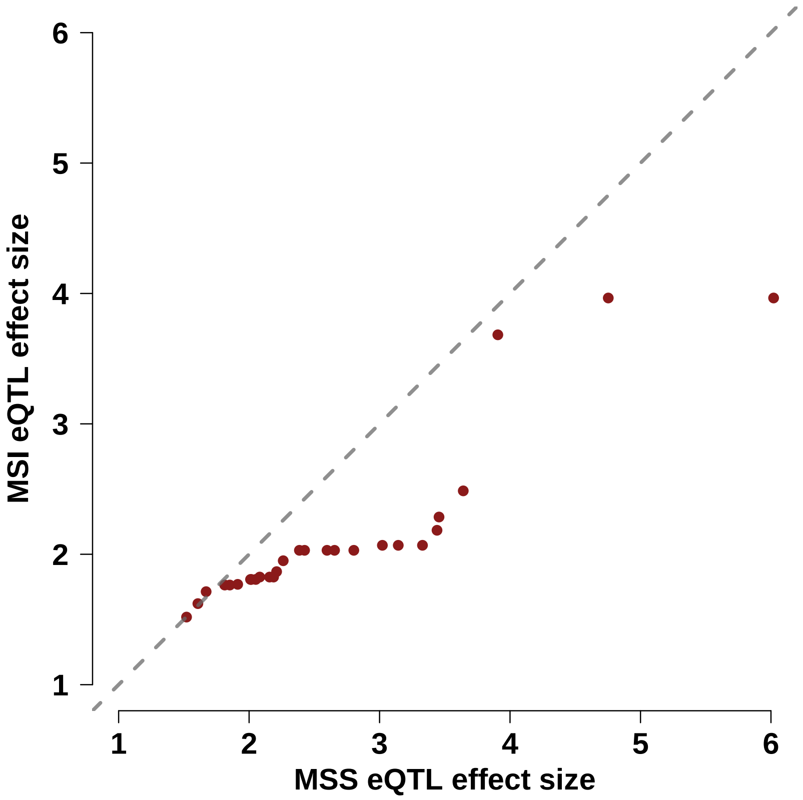

Supplementary Figure S17: QQ plot comparing the quantiles of MSS and MSI significant mutation eQTL effect sizes.

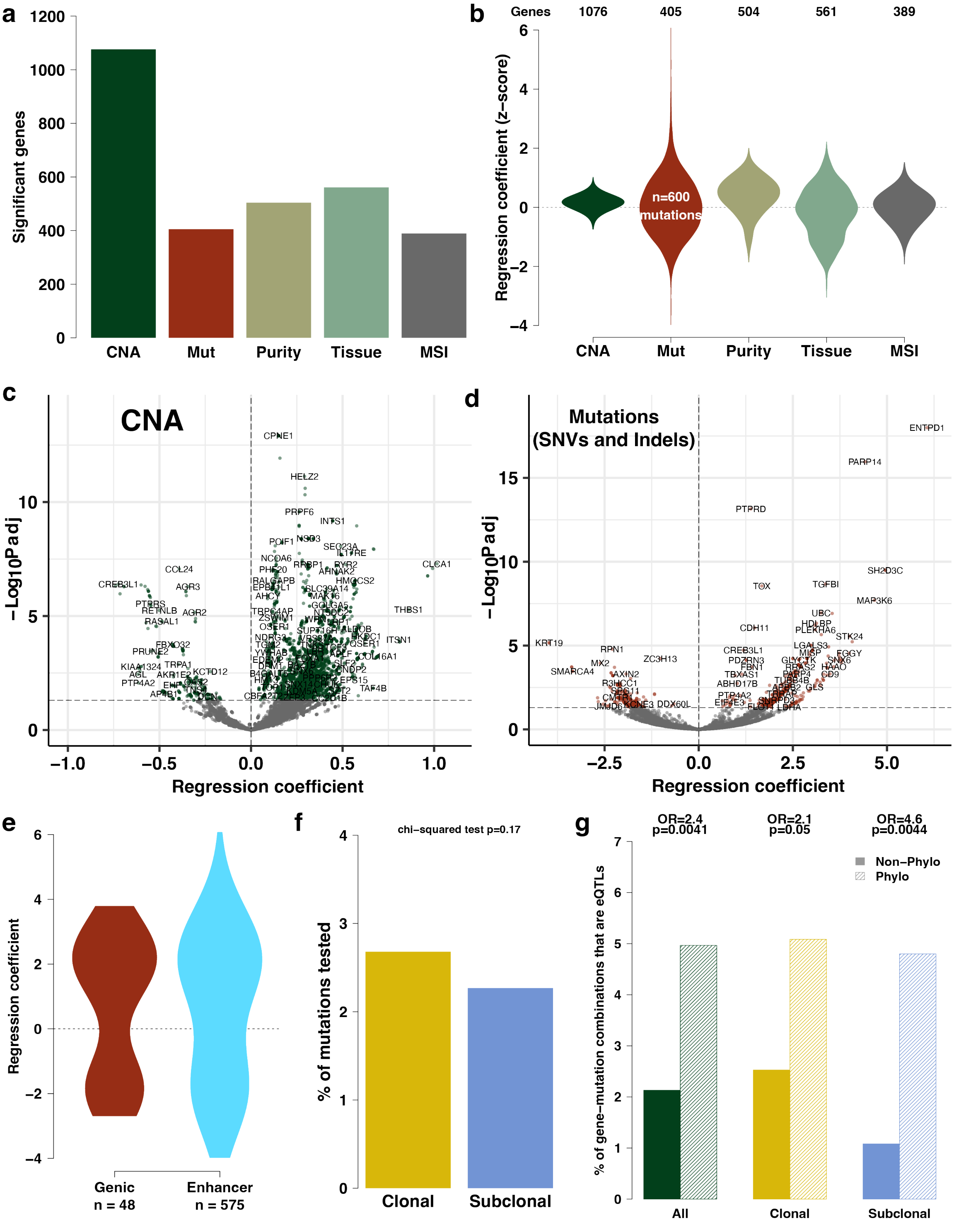

Supplementary Figure S18: Genetic control of expression with eQTL analysis with MSI status added as a cofactor. **(a)** The number of genes with significant models for each data type. **(b)** The distribution of regression coefficients (effect sizes) for each data type. **(c)** and **(d)** Volcano plots highlighting selected genes that were significant for CNA and Mut eQTLs respectively (linear regression two-sided t-tests, p-values are FDR-adjusted). **(e)** In comparison to non-synonymous SNVs (NS), enhancer (Enh) mutations tended to have large effect sizes and a higher proportion of positive effect sizes. **(f)** The proportion of subclonal mutations that were associated with detectable changes in cis gene expression tended to be lower than for clonal eQTL mutations (two-sided chi-squared test). **(g)** Visualisation of two-sided Fisher's exact tests showing that gene-mutation combinations were more likely to be eQTLs if they were associated with recurrent phylogenetic genes (genes found to be phylogenetic in at least 3 tumours) for subclonal mutations and that this was not significant for clonal mutations. P-values are not corrected for multiple testing.

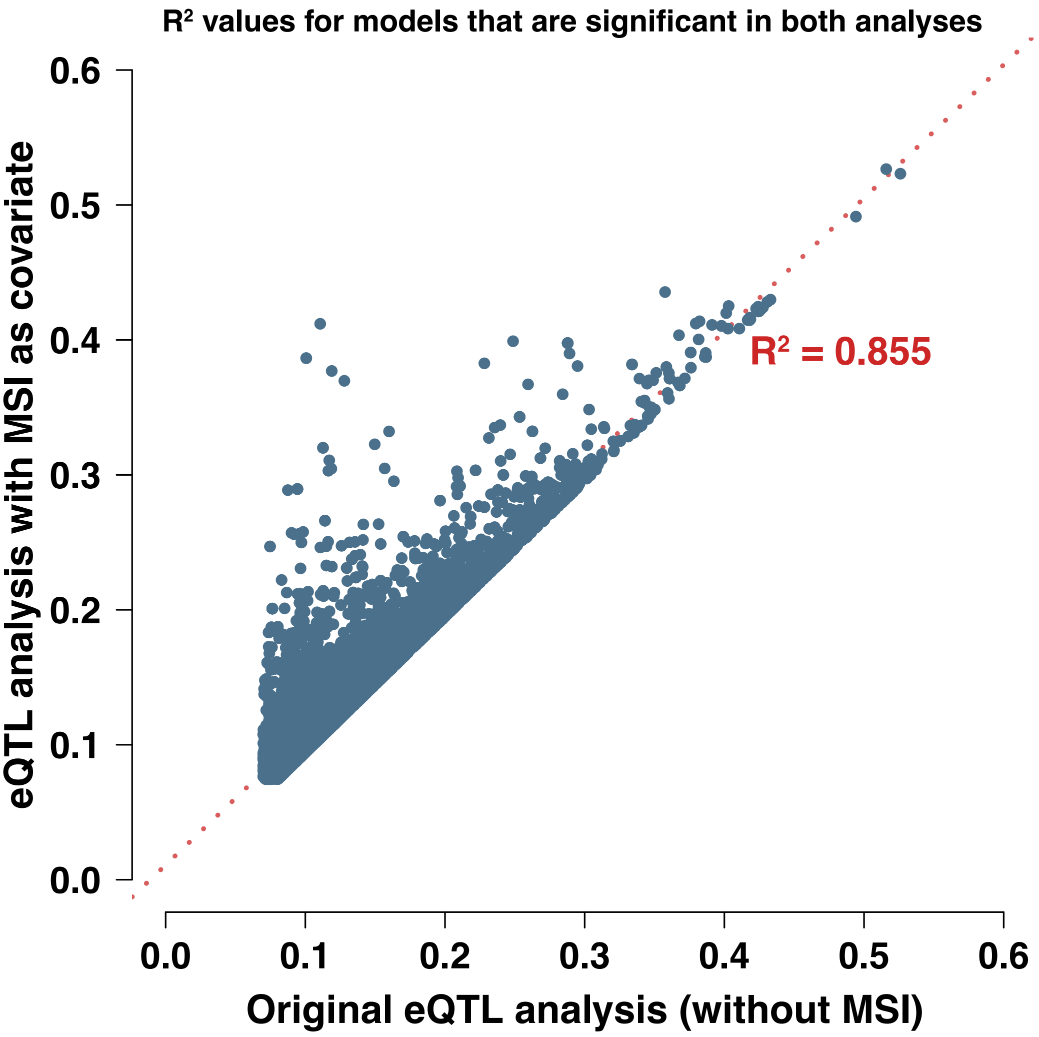

Supplementary Figure S19: Scatter plot showing correlation of R^2 values for models significant in both the original eQTL analysis and the analysis with MSI included as a covariate.

6 Mutations and phylogenetics

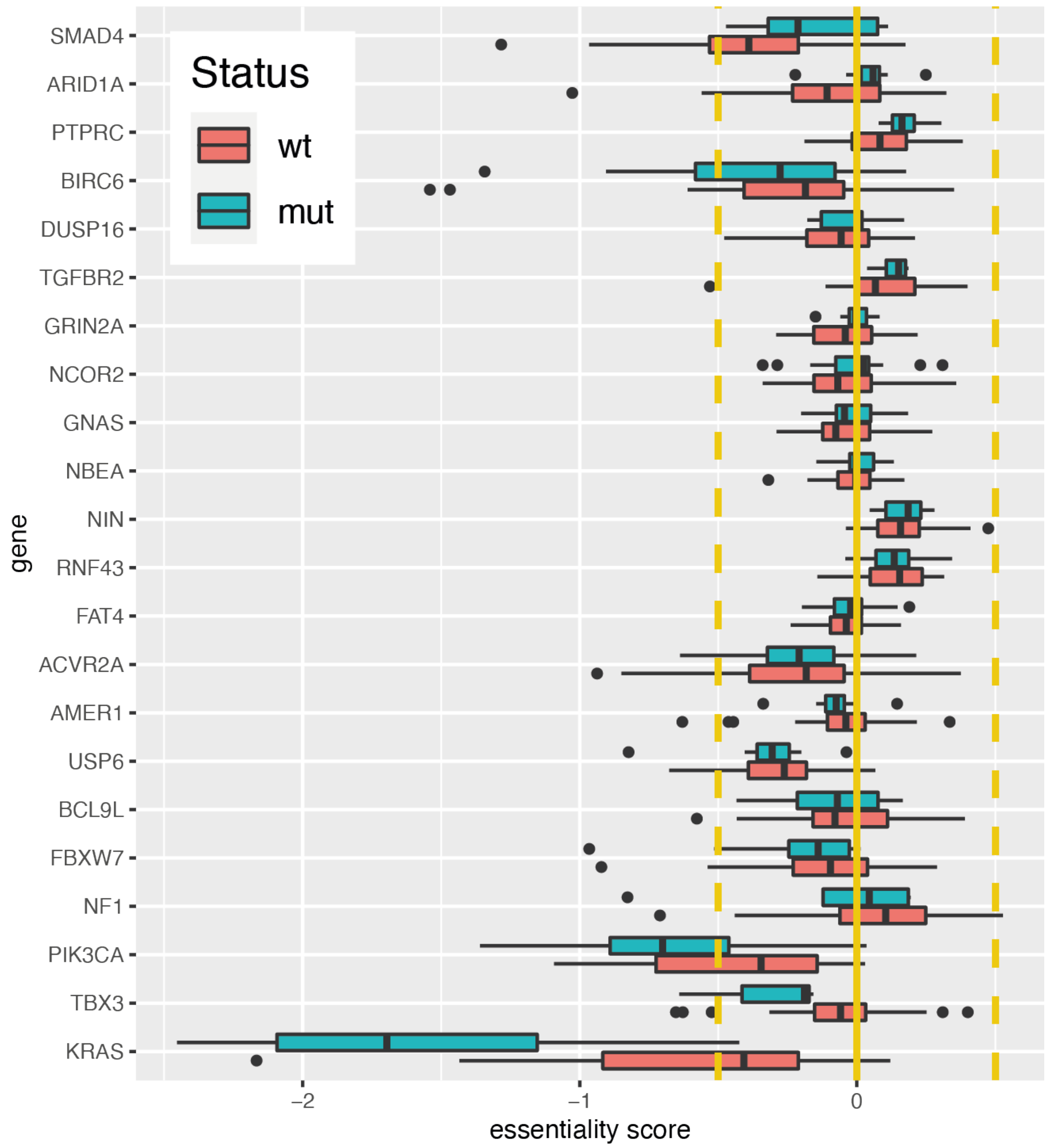

Supplementary Figure S20: Gene essentiality analysis with CRISPR screens. Essentiality scores from the cancer dependency map (DepMap) of many putative driver genes we find subclonal are not significant across hundreds of cancer cell lines, and are not significantly different across mutant versus wild-type subpopulations of cell-lines (which would be consistent with oncogenic function), with the exception of KRAS and PIK3CA. Orange lines denote the significance thresholds of the essentiality score as defined by DepMap, i.e. half of the median essentiality scores observed for prior known essential genes (= -1). The lower and upper hinges of the boxes show the first and third quartiles, the whiskers extend to the most extrem values up to 1.5 inter quartile ranges from the whisker and values outside of this range are shown as individual points. The number of mutated and unmutated models per gene are: 26+10 KRAS, 4+13 TBX3, 19+27 PIK3CA, 31+72 NF1, 24+14 FBXW7, 21+3 BCL9L, 22+60 USP6, 12+0 AMER1, 21+7 ACVR2A, 66+31 FAT4, 14+32 NIN, 34+107 NBEA, 29+25 GNAS, 18+39 NCOR2, 13+36 GRIN2A, 10+5 TGFBR2, 7+8 DUSP16, 34+70 BIRC6, 17+54 PTPRC, 19+15 ARID1A and 11+8 SMAD4.

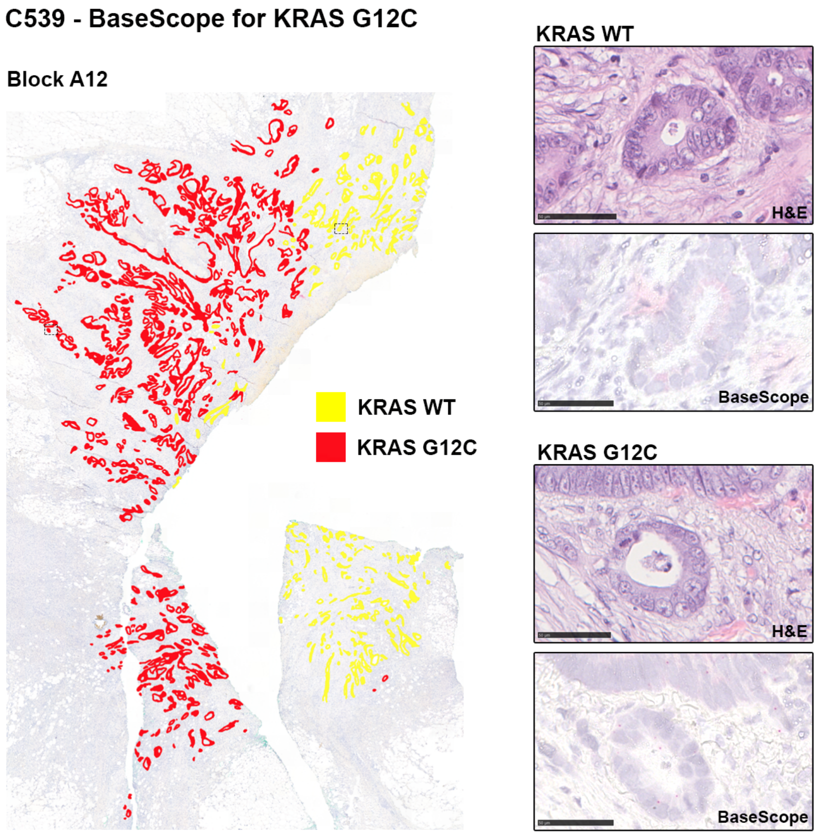

Supplementary Figure S21: High resolution figure image of in situ mutation detection with BaseScope® for the KRAS G12C subclonal variant in C539. H&E and BaseScope® staining was performed once, and scale bars are 50 µm.

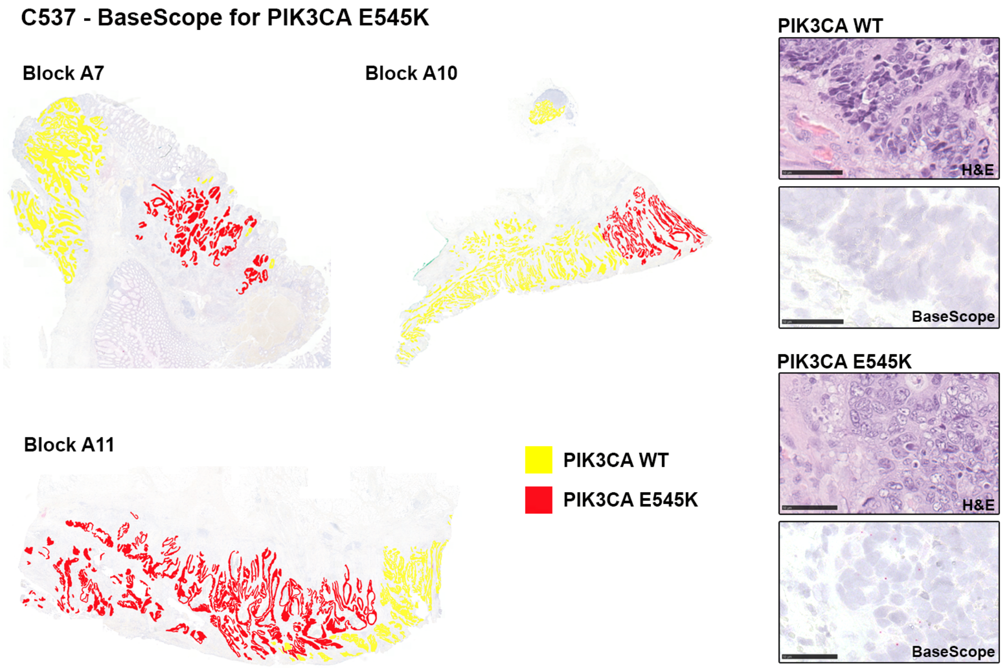

Supplementary Figure S22: High resolution figure image of in situ mutation detection with BaseScope® for the PIK3CA E545K subclonal variant in C537. H&E and BaseScope® staining was performed once, and scale bars are 50 µm.

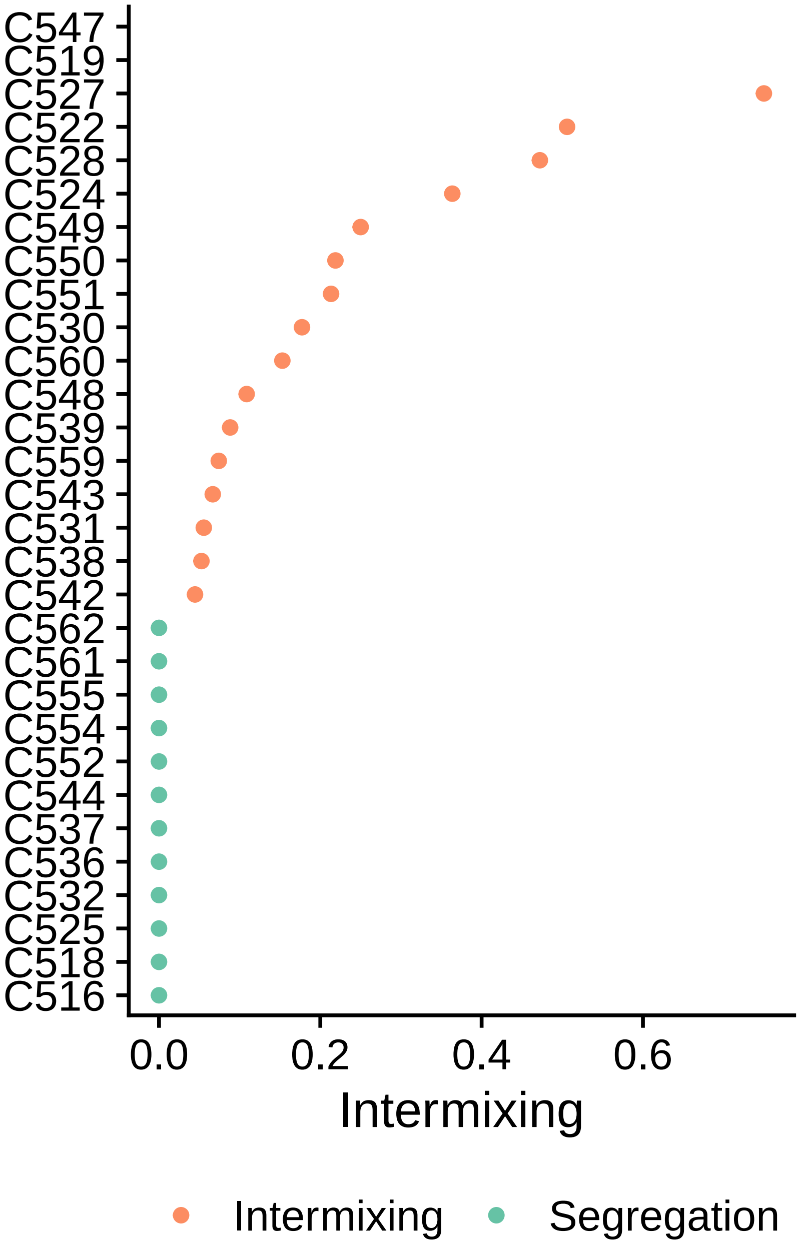

Supplementary Figure S23: Measurements of subclonal intermixing for each patient. Reported values for each tumour are computed as specified in Methods section 4.

7 Selection inference

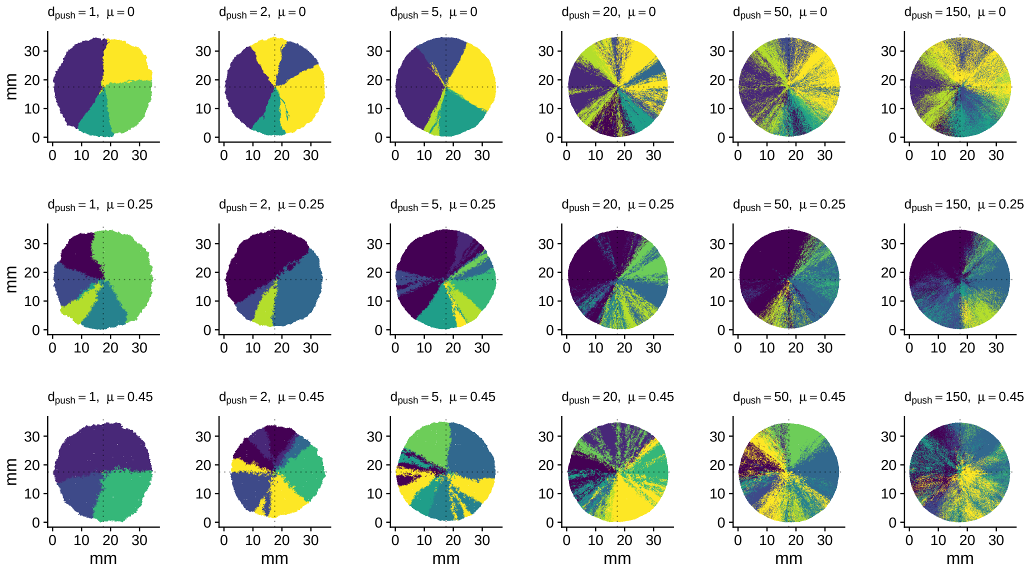

Supplementary Figure S24: Simulations of tumour growth with different mutation rate and peripheral growth parameters. Colours indicates clonal lineages

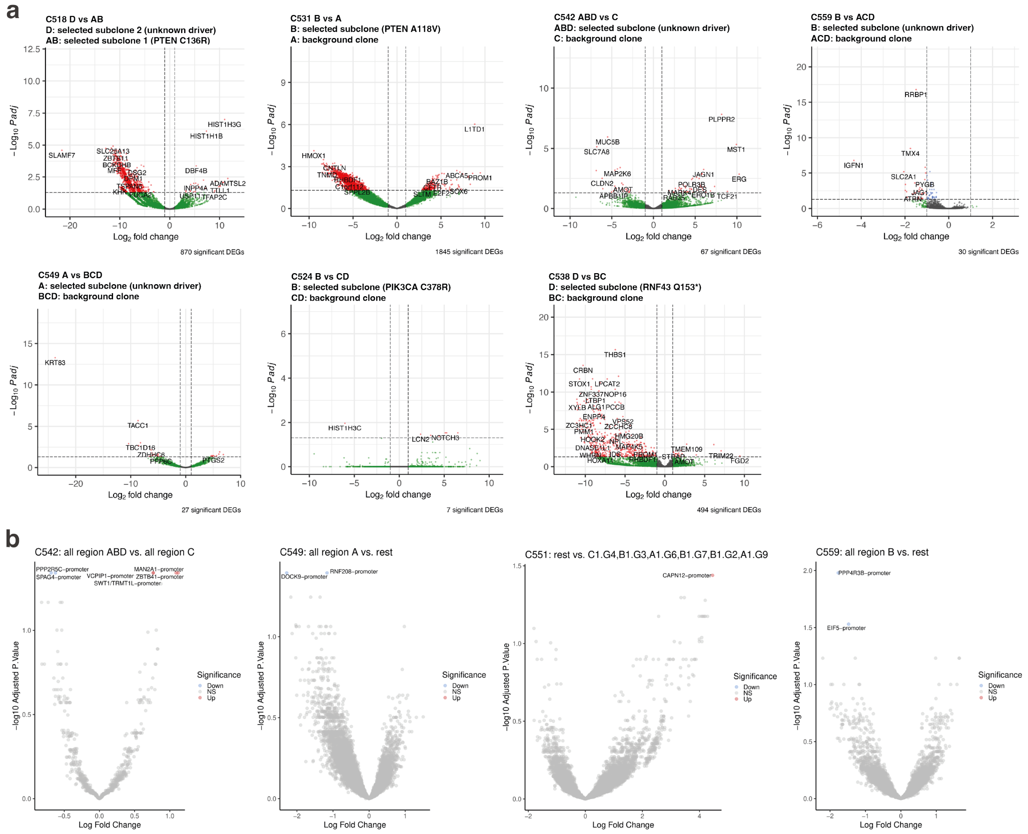

Supplementary Figure S25: Differential gene expression and chromatin accessibility between detected subclone and background clone. **(a)** Differential gene expression between the subclone and background clone for all tumours with selection (AIC) for which there was sufficient samples (linear regression two-sided t-test, p-values are adjusted for multiple comparisons). **(b)** Differential ATAC peak between subclone and background clone for those tumours with no detected genetic driver (linear regression two-sided t-test, p-values are adjusted for multiple comparisons).

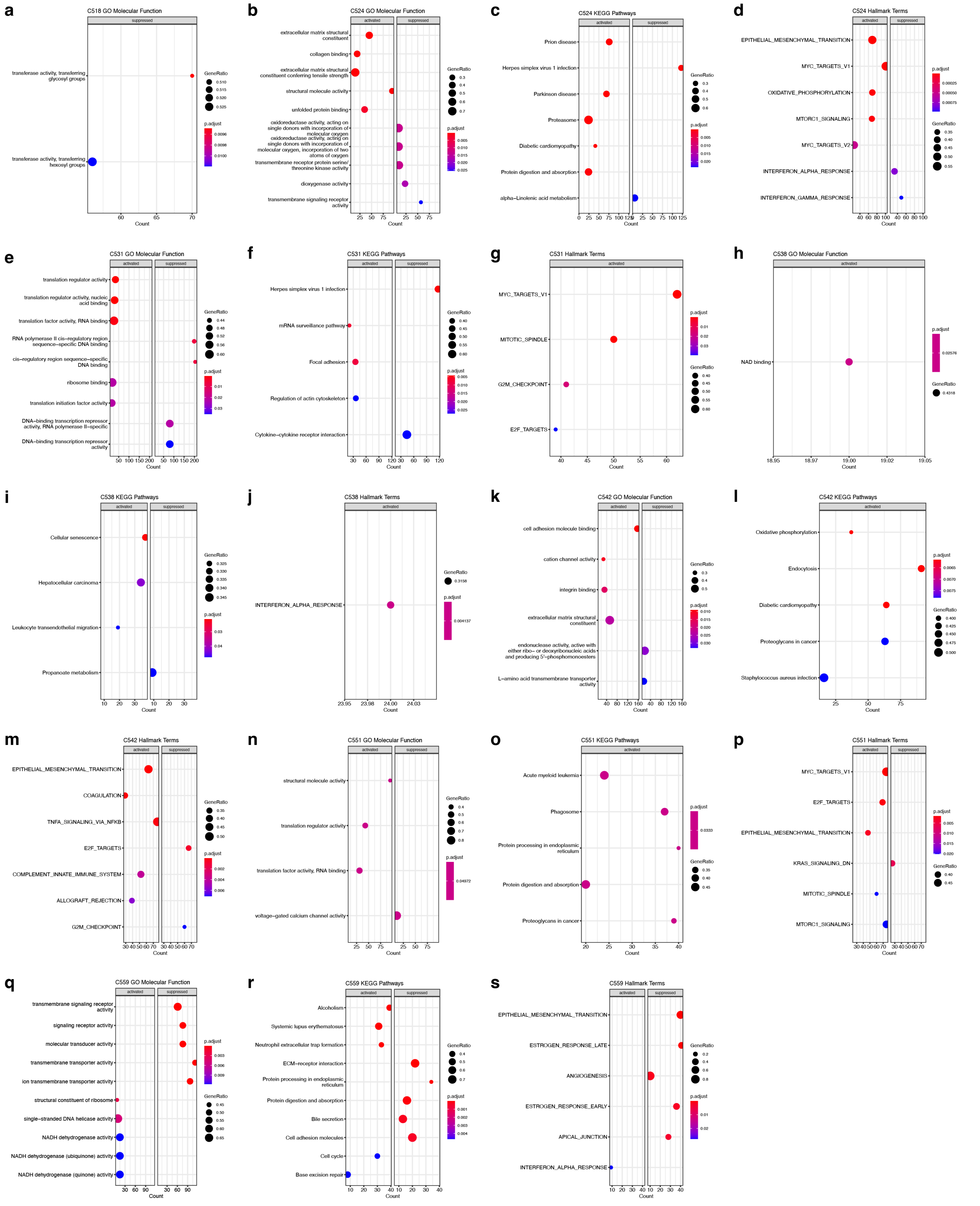

Supplementary Figure S26: Gene set enrichment analysis based on gene expression of subclone versus background clone. Enrichment tests were two-sided and p-values are adjusted for multiple comparisons. **(a)** C518. **(b-d)** C524. **(e-g)** C531. **(h-j)** C538. **(k-m)** C542. **(n-p)** C551. **(q-s)** C559.

8 Combining analyses

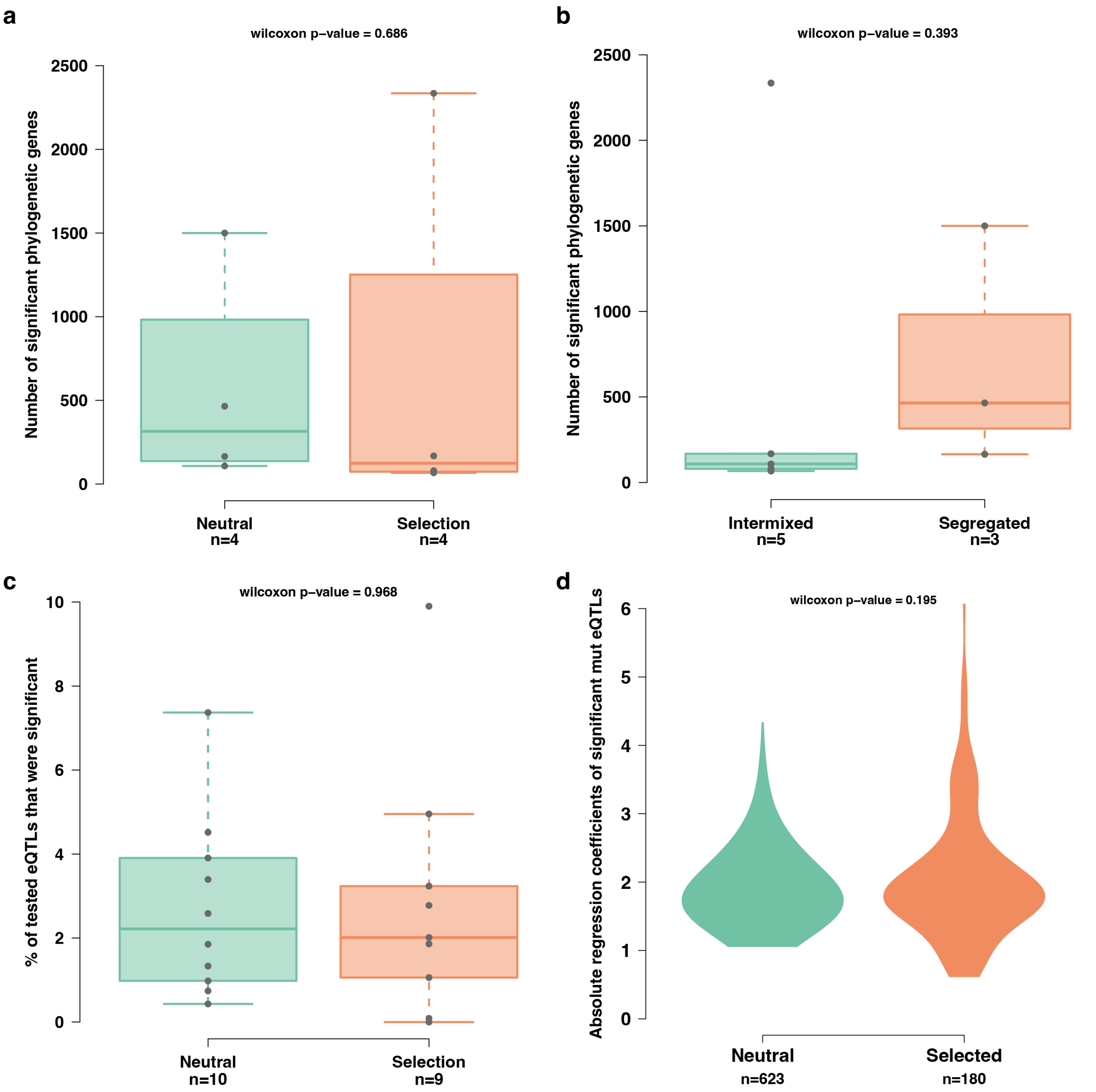

Supplementary Figure S27: Assessment of heritable changes associated with subclonal selection. All tests are two-sided Wilcoxon signed-rank tests. **(a)** No association between the number of phylogenetic genes and the presence of subclone selection. **(b)** No association between the number of phylogenetic genes and spatial segregation/intermixing. **(c)** No association between the percentage of tested eQTL genes that were significant and the presence of subclone selection. **(d)** No association between the magnitude of heritable gene expression changes and the presence of subclone selection.
