# Supplementary material for: Phenotypic plasticity and genetic control in colorectal cancer evolution
Source: Nature. 2022 Oct 26;611(7937):744–53. doi: 10.1038/s41586-022-05311-x (PMC9684078; doi:10.1038/s41586-022-05311-x)
Supplement: Supplementary file 2 — Reporting Summary [file 41586_2022_5311_MOESM2_ESM.pdf]

nature portfolio

Corresponding author(s): Trevor A Graham
Andrea Sottoriva

Last updated by author(s): Aug 5, 2022

Reporting Summary

Nature Portfolio wishes to improve the reproducibility of the work that we publish. This form provides structure for consistency and transparency in reporting. For further information on Nature Portfolio policies, see our Editorial Policies and the Editorial Policy Checklist.

Statistics

For all statistical analyses, confirm that the following items are present in the figure legend, table legend, main text, or Methods section.

n/a	Confirmed	
☐	☒	The exact sample size (*n*) for each experimental group/condition, given as a discrete number and unit of measurement
☐	☒	A statement on whether measurements were taken from distinct samples or whether the same sample was measured repeatedly
☐	☒	The statistical test(s) used AND whether they are one- or two-sided<br>*Only common tests should be described solely by name; describe more complex techniques in the Methods section.*
☐	☒	A description of all covariates tested
☐	☒	A description of any assumptions or corrections, such as tests of normality and adjustment for multiple comparisons
☐	☒	A full description of the statistical parameters including central tendency (e.g. means) or other basic estimates (e.g. regression coefficient) AND variation (e.g. standard deviation) or associated estimates of uncertainty (e.g. confidence intervals)
☐	☒	For null hypothesis testing, the test statistic (e.g. *F*, *t*, *r*) with confidence intervals, effect sizes, degrees of freedom and *P* value noted<br>*Give P values as exact values whenever suitable.*
☐	☒	For Bayesian analysis, information on the choice of priors and Markov chain Monte Carlo settings
☐	☒	For hierarchical and complex designs, identification of the appropriate level for tests and full reporting of outcomes
☐	☒	Estimates of effect sizes (e.g. Cohen's *d*, Pearson's *r*), indicating how they were calculated

Our web collection on statistics for biologists contains articles on many of the points above.

Software and code

Policy information about availability of computer code

Data collection	No software was used for data collection
Data analysis	https://github.com/sottorivalab/EPICC2021_data_analysis<br>https://github.com/sottorivalab/EPICC2021_data_analysis_RNA<br>https://github.com/T-Heide/MLLPT

For manuscripts utilizing custom algorithms or software that are central to the research but not yet described in published literature, software must be made available to editors and reviewers. We strongly encourage code deposition in a community repository (e.g. GitHub). See the Nature Portfolio guidelines for submitting code & software for further information.

Data

Policy information about availability of data

All manuscripts must include a data availability statement. This statement should provide the following information, where applicable:

- Accession codes, unique identifiers, or web links for publicly available datasets
- A description of any restrictions on data availability
- For clinical datasets or third party data, please ensure that the statement adheres to our policy

This manuscript is a reanalysis of data in associated manuscript Heide, Househam et al. (2022). The data in that associated manuscript are available on Mendeley (DOI 10.17632/7wx3chtsxx.1) and sequence data have been deposited at the European Genome-phenome Archive (EGA), which is hosted by the EBI and the CRG, under accession number EGAS00001005230. Additional supplementary information is available on figshare (DOI 10.6084/m9.figshare.c.6122193).

March 2021

Field-specific reporting

Please select the one below that is the best fit for your research. If you are not sure, read the appropriate sections before making your selection.

☒ Life sciences ☐ Behavioural & social sciences ☐ Ecological, evolutionary & environmental sciences

For a reference copy of the document with all sections, see nature.com/documents/nr-reporting-summary-flat.pdf

Life sciences study design

All studies must disclose on these points even when the disclosure is negative.

Sample size	No power calculation was performed prior to starting the study. Post hoc power calculations were performed throughout the study and results interpreted in light of these analyses.
Data exclusions	Various data exclusions were performed on an analysis-by-analysis basis (usually due to available sample numbers with required multi-omics and/or samples per tumour). These are specified in the manuscript at the point that each analysis is described.
Replication	Specific replication on individual samples was not possible due to insufficient material (as described in associated manuscript detailing wet-lab data generation). Repeat sampling of tumours provided a level of psuedo-replication.
Randomization	Randomization was not relevant to this study. The explicit analysis clinical-pathological of covariates, such as mismatch repair status, is reported in the manuscript. Our study was an observational cohort study so randomization was not possible. No differential outcomes were examined so randomization was not needed.
Blinding	Researchers were blinded to clinical-pathological data.

Reporting for specific materials, systems and methods

We require information from authors about some types of materials, experimental systems and methods used in many studies. Here, indicate whether each material, system or method listed is relevant to your study. If you are not sure if a list item applies to your research, read the appropriate section before selecting a response.

Materials & experimental systems

n/a	Involved in the study	
☒	☐	Antibodies
☒	☐	Eukaryotic cell lines
☒	☐	Palaeontology and archaeology
☒	☐	Animals and other organisms
☒	☐	Human research participants
☒	☐	Clinical data
☒	☐	Dual use research of concern

Methods

n/a	Involved in the study	
☒	☐	ChIP-seq
☒	☐	Flow cytometry
☒	☐	MRI-based neuroimaging
